# Patient Identification Based on Deep Metric Learning for Preventing Human Errors in Follow-up X-Ray Examinations

**DOI:** 10.1007/s10278-023-00850-9

**Published:** 2023-06-12

**Authors:** Yasuyuki Ueda, Junji Morishita

**Affiliations:** 1 Department of Medical Physics and Engineering, Area of Medical Imaging Technology and Science, Graduate School of Medicine, Division of Health Sciences, Osaka University, Osaka, Japan; 2 Department of Health Sciences, Faculty of Medical Sciences, Kyushu University, Fukuoka, Japan

**Keywords:** Chest X-ray image, Patient identification, Patient verification, Biometrics, Deep metric learning

## Abstract

Biological fingerprints extracted from clinical images can be used for patient identity verification to determine misfiled clinical images in picture archiving and communication systems. However, such methods have not been incorporated into clinical use, and their performance can degrade with variability in the clinical images. Deep learning can be used to improve the performance of these methods. A novel method is proposed to automatically identify individuals among examined patients using posteroanterior (PA) and anteroposterior (AP) chest X-ray images. The proposed method uses deep metric learning based on a deep convolutional neural network (DCNN) to overcome the extreme classification requirements for patient validation and identification. It was trained on the NIH chest X-ray dataset (ChestX-ray8) in three steps: preprocessing, DCNN feature extraction with an EfficientNetV2-S backbone, and classification with deep metric learning. The proposed method was evaluated using two public datasets and two clinical chest X-ray image datasets containing data from patients undergoing screening and hospital care. A 1280-dimensional feature extractor pretrained for 300 epochs performed the best with an area under the receiver operating characteristic curve of 0.9894, an equal error rate of 0.0269, and a top-1 accuracy of 0.839 on the PadChest dataset containing both PA and AP view positions. The findings of this study provide considerable insights into the development of automated patient identification to reduce the possibility of medical malpractice due to human errors.

## Introduction

Patient misidentification due to human error remains a major concern in the healthcare industry [1–8]. According to a previous study [3], the average rate of misfiled cases for radiography at a hospital is 0.075%.

A previous paper [5] reported that “a 2006 study [6] reviewing several adverse events databases in the United States estimated that between 1,300 and 2,700 adverse events relating to wrong side or site, wrong procedure, and wrong patient occurred annually. It identified radiology as the second most common hospital department (after the operating room) to perform wrong-site procedures.” Although the primary risk of patient misidentification in clinical X-ray examinations is radiation exposure of the wrong patient, it can lead to serious incidents or accidents, such as operating of the wrong patient [6].

Risk management in clinics and hospitals employs a standard process for patient verification because confirming patient-specific information (such as the full name and date of birth) is critical [9]. Although using two patient identifiers can improve the reliability of patient identification, such a process is not the most effective for preventing human errors, resulting in misfiling and misidentification [10, 11]. To minimize the risk of human error and improve healthcare workflow and utility, clinics and hospitals may use biometrics, such as fingerprints and face recognition, for routine examinations. However, the use of these methods can increase the workload of healthcare providers.

Digital transformation in healthcare can be realized using various technologies to streamline operations for healthcare providers and ensure patient safety. Several types of clinical images, such as chest X-ray or scout images, exhibit considerable potential for use in biometric patient verification [1–3, 12–27] and forensic personal identification [28–35]. The potential risk of patient registration errors owing to multiple factors, including human errors, can be reduced by consolidating clinical information and using it for patient identity verification. Furthermore, such a technology has the potential to identify the personal information of a patient without the patient verbally stating their name or date of birth. Several biometric methods based on clinical images have been proposed to achieve satisfactory patient verification and identification [1–3, 12–24].

Deep learning-based technologies have been applied to several tasks using clinical X-ray images [36–39] of the chest [12, 28, 36, 40–46], including lesion detection and estimation of individual attributes, such as gender and age. Some issues need to be addressed for deep learning-based biometric systems to be utilized in clinics or hospitals. In hospitals and clinics, a biometric system can identify a large number of patients during the second examination without retraining the system for each new patient. Several methods have been reported for the classification of chest X-ray images used in clinics or hospitals [12]; however, such methods are yet to be used in actual clinical applications.

The performance of such biometric techniques applied to clinics and hospitals can degrade owing to variabilities in the clinical chest X-ray images, including variations in posteroanterior (PA) and anteroposterior (AP) view positions, image processing, and patient conditions (pre- and post-operative or trauma) [13, 14]. In particular, regarding view positions, healthy subjects are examined in the standing and PA view positions in routine chest X-ray imaging. However, depending on the patient’s condition (such as inability to stand or postmortem), the examination is performed in the spine or in the sitting AP view position. Previous studies have revealed that classification between PA and AP view positions is possible in chest X-ray images [36]; this also implies that there is a domain-shift problem between PA and AP view positions. Therefore, differences in the positions of PA and AP views, including differences in the patient’s condition and imaging geometry, can have a significant impact on their biometric performance. Although some studies have addressed this problem [28], the domain-shift problem caused by differences in view position has not been resolved, and concerns regarding its clinical application are yet to be addressed. Biometric systems for clinics and hospitals are expected to not only have adequate biometric performance based on clinical images [47–49] but also exhibit robustness under clinical image variabilities; therefore, such a biometric system, which exhibits good performance under varied view positions, will potentially be effective for use in clinics and hospitals.

The objective of the study was to (a) develop a deep learning-based feature extractor and classifier using the similarity index to perform patient identity verification during chest X-ray examination and (b) quantitatively evaluate the outcomes of the proposed method on clinical chest X-ray datasets with clinical variabilities, such as different view positions.

## Materials and Methods

### Outline of the Proposed Method

**Fig. 1:**
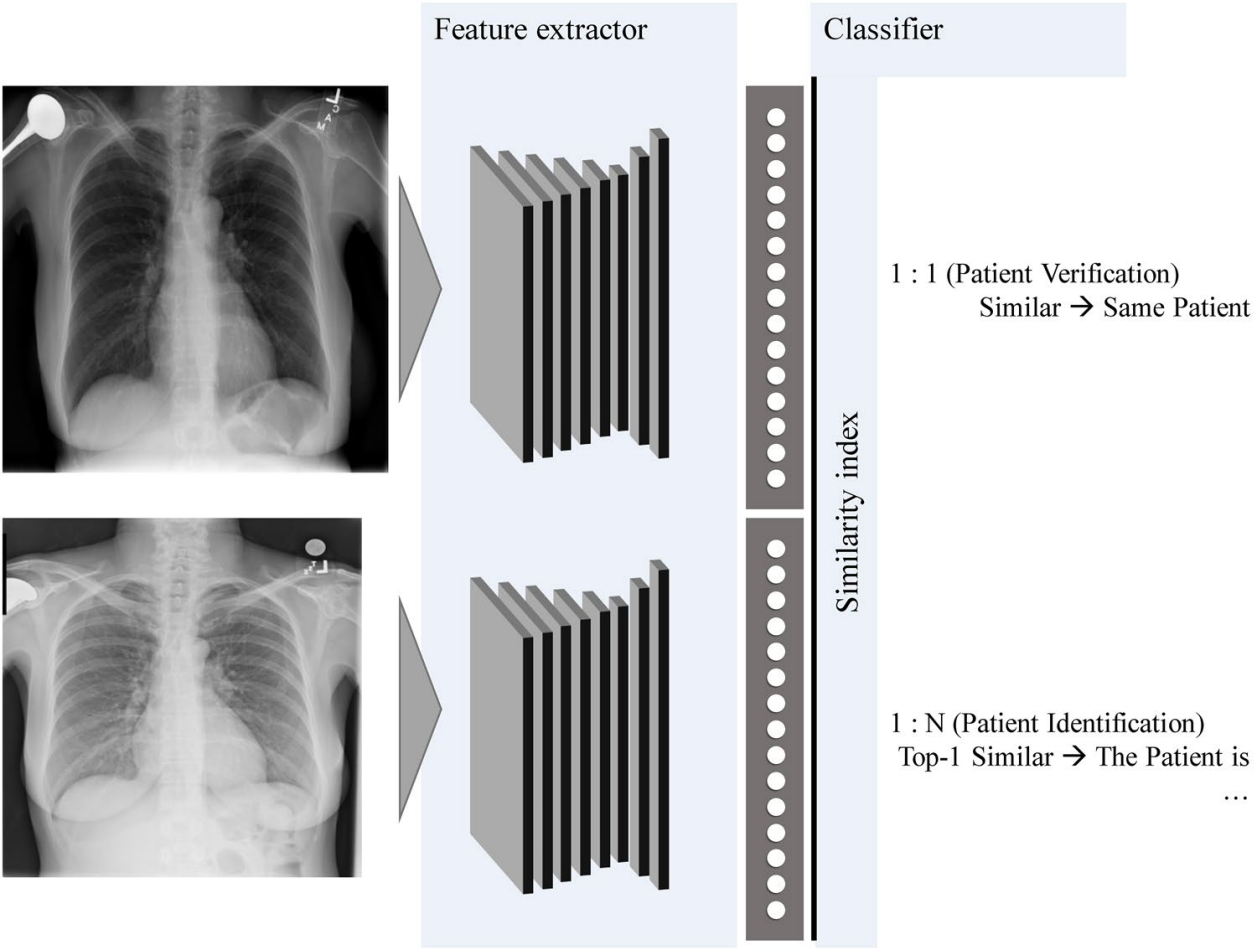
Proposed system for patient verification and identification

The proposed deep learning metric-based patient verification and identification system is illustrated in Fig. 1. The system comprises three units: (i) image acquisition and preprocessing, (ii) feature extraction, and (iii) identification. The first unit acquires an image with a routine chest X-ray examination and performs image postprocessing. Healthcare providers are not required to perform any additional work in biometric patient verification. In the second unit, the feature extractor trained by the proposed model extracts the features that identify an individual from chest X-ray images. In the final unit, the similarity index of the extracted features between clinical chest X-ray images and stored clinical datasets is used to determine whether the patients are the same.

The proposed method was evaluated using four test datasets different from the dataset used for training and validation. Two datasets were public datasets, whereas the other two were clinical chest X-ray image datasets.

## Datasets

The experiment was a retrospective, observational study and was approved by the Institutional Review Boards at Osaka University (Approval Number: 19422–6). Therefore, informed consent was not mandatory. All procedures performed in this study conformed to the guidelines and ethics set by the Helsinki Declaration.

Table 1 summarizes the patient demographics in the five datasets used for training, validating, and testing the DCNN used in the proposed method. The ChestX-Ray8 dataset [50], used for training and validation, included 57,452 chest X-ray images from 3,245 patients with at least eight chest X-ray images per patient that were examined on both PA and AP view positions. All 57,452 chest X-ray images were used in either training or validating subsets, but not both. Of the 57,452 chest X-ray images of 3,245 patients, 50,962 images were used for training and 6,490 images for validating the trained model. To enable mini-batch learning within the computation limitations for training and validation, the image pixel spacing of all the images was resampled to 1.0 × 1.0 mm^2^ (276 to 495 and 261 to 495 pixels for rows and columns, respectively) using bicubic interpolation and by cropping the central 320 × 320 pixels.

**Table 1:** Datasets used for DCNN training, validation, and testing

Public dataset	ChestXray8	CheXpert	PadChest	
Number of patients	3,245	10,224	6,376	Patients
Number of images	57,452	32,972	19,713	Images
Gender				Patients
Male	1,892	5,272	3,594	
Female	1,353	4,952	2,780	
Others	0	0	2	
View position				Images
PA	26,018	6	7,867	
AP	31,434	32,966	11,846	
Age categories				Patients
Neonate, infant (0 years)	0		152	
Young child (1–4 years)	0		222	
Older child (5–10 years)	0		85	
Adolescent (11–19 years)	0		62	
Adult (20 years or more)	3,245		5,855	
Examination interval categories (from baseline to follow-up X-ray examination dates)				Image–pairs
Under 1 year			10,559	
From 1 to 5 years			2,241	
Over 5 years			537	

Clinical dataset	ML	YU	
Number of images	81,109	25,547	images
Number of patients	31,366	12,517	patients
Examined baseline X-rays	12,790	8,417	
Examined baseline and follow-up X-rays	18,576	4,100	
Age (at the baseline examination)			years
Mean ± SD	55 ± 15	64 ± 16	
Range	15–95	20–100	
Gender			patients
Male	13,845	6,653	
Female	17,521	5,864	
View position			images
PA	81,109	23,953	
AP	0	1,594	
Subsets of clinical dataset (with at least two examinations per patient)			
Number of images	68,319	17,130	images
Number of patients	18,576	4,100	patients
Pair of view positions (baseline and follow-up)			pairs
PA–PA	49,743	11,487	
AP–PA	0	613	
PA–AP	0	306	
AP–AP	0	624	
Examination intervals between the baseline and follow-up X-ray examination dates			days
Mean ± SD	1,051 ± 635	100 ± 115	
Range	0–2,932	0–489	

*ML* Morishita Laboratory, *YU* Yamaguchi University, *PA* Posteroanterior, *AP* Anteroposterior, *SD* Standard Deviation, *PA–PA* subset for PA view at both examinations, *AP–PA* subset for AP view at baseline and PA view at follow–up examinations, *PA–AP* subset for PA view at baseline and AP view at follow–up examinations, *AP–AP* subset for AP view for both examinations

Two public datasets and two clinical datasets were used for testing. The first public dataset, PadChest [51], included 19,713 chest X-ray images from 6376 patients with at least two PA or AP chest X-ray images per patient. The image pixel spacing of all images was resampled to 1.0 × 1.0 mm^2^ (151 to 431 and 158 to 431 pixels for rows and columns, respectively) using bicubic interpolation. Furthermore, the bit depth was rescaled linearly down to 8 bits. The second public dataset, CheXpert [52], included 32,972 chest X-ray images from 10,224 patients. We used the CheXpert-V1.0-Small dataset in which the image pixel size and bit depth of all images were 512 × 512 pixels and 8 bits, respectively.

The first clinical dataset named “Morishita Laboratory (ML)” comprised 81,109 chest X-ray images from 31,366 patients who underwent chest X-ray screening examinations between March 1986 and October 2004 in Iwate, Japan. Images of patients in the ML dataset were acquired using a computed radiography system (Fujifilm, Tokyo, Japan) under a matrix size of 1760 × 1760 pixels, pixel spacing of 0.2 × 0.2 mm^2^, and 10-bit greyscale. The second clinical dataset named “Yamaguchi University (YU)” comprised 25,547 chest X-ray images from 12,517 patients obtained by random sampling from patients aged 20 years or older who underwent routine chest X-ray examinations between October 2015 and March 2017 at Yamaguchi University Hospital, Japan. All the images in the YU dataset were acquired using a flat-panel detector system (AeroDR; Konica Minolta Inc., Tokyo, Japan) or a computed radiography system (REGIUS MODEL 110; Konica Minolta Inc., Tokyo, Japan) with a matrix size of 2430 × 1994 pixels, pixel spacing of 0.175 × 0.175 mm^2^, and 12-bit greyscale. The data were processed using the diagnostic imaging workstation (model CS-7 version 1.20–1.30; Konica Minolta Inc., Tokyo, Japan). In addition to the public dataset, we resampled all the images in the two clinical datasets to images with a pixel spacing of 1.0 × 1.0 mm^2^ using bicubic interpolation. Furthermore, the bit depth was rescaled linearly down to 8 bits.

## DCNN Learning

### Outline of DCNN Learning by the Proposed Model

The EfficientNetV2-S backbone network with metric learning was used to train the DCNN for classifying individual examined patients based on chest X-ray images. Deep learning was performed using a computer with a GeForce 3090 Ti (NVIDIA, Santa Clara, Calif) graphics processing unit, Core i9-10900X 3.70 GHz (Intel, Corp., Santa Clara, Calif) central processing unit, and 128 GB of random-access memory.

Python 3.10.8 and PyTorch 1.13.0 + cu117 were used to perform DCNN training, validation, and testing using the element-wise adaptive sharpness-aware minimization optimizer [53] (base optimizer, SGD with a momentum of 0.8, and a weight decay of 0.0005) with a neighborhood size, rho, of 2.0, batch size of 60, smooth cross-entropy loss function with a label smoothing of 0.1, and cosine-decay learning rate scheduler (initial–last: 0.02–0.0004) without warmup. During each training epoch, each image was augmented to correspond to the variabilities in clinical chest X-ray images as follows: random rotation between − 10° and 10° and random perspective transformation with a probability of 0.1 and distortion scale of 0.25 with bicubic resampling. The training time of the proposed method was approximately 70 h for 300 epochs of learning.

## Feature Extractor Used by the Proposed Model

The EfficientNetV2-S backbone network with metric learning was used to train the DCNN for classifying individual examined patients based on chest X-ray images. EfficientNet uses mobile inverted bottleneck convolution (MBConv), similar to MobileNetV2 [54] and MnasNet [55]. A compound scaling method that uniformly scales each dimension with a fixed set of scaling coefficients is incorporated in the system [56]. In EfficientNetV2, fused-MBConv layers replace the 3 × 3 depth-wise convolution (conv3 × 3), and the 1 × 1 boosted convolution (conv1 × 1) is expanded in MBConv in the original EfficientNet with a normal 3 × 3 convolution to improve training performance [57]. The output values of the feature extractor trained using the proposed method were used as features to characterize individuals.

## Classifier Used by the Proposed Model

Conventional image classifiers cannot achieve high generalizability even with deep learning and require numerous training samples of medical images and a long training time, which degrades the performance of the DCNN. By contrast, deep metric learning is a technique for improving the accuracy of classifiers [58]. Deep metric learning can be used to develop a feature extractor with high generalizability using fewer classes and images [58].

**Fig. 2:**
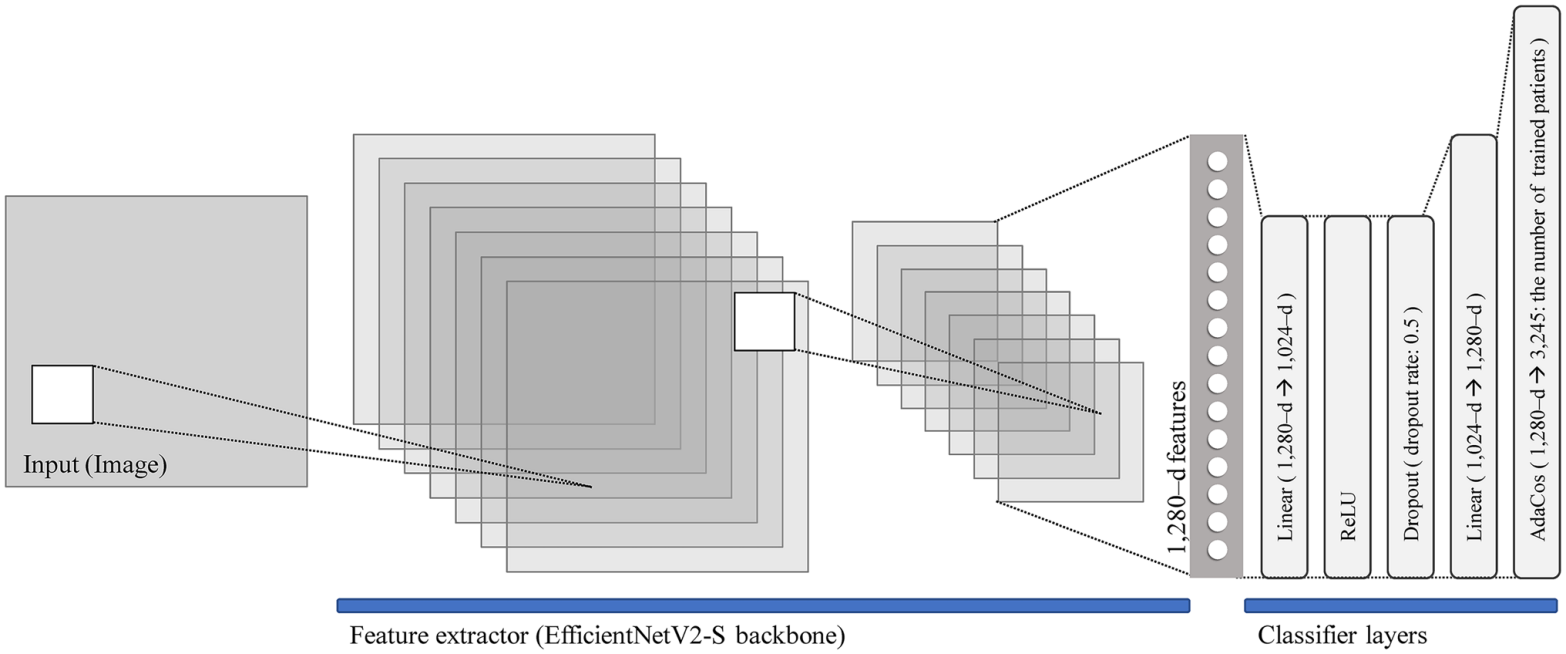
Overview of the DCNN used in the proposed method. EfficientNetV2-S backbone is used as the feature extractor, and the classifier network consists of a linear layer, ReLU activation function, dropout layer, linear layer, and AdaCos [59] in that order. ReLU: rectified linear unit

Figure 2 displays the classifier used by the proposed method that converts the features in the feature extractor output into the prediction of the 3245 classes of patients for the training subset. The classifier network comprises a nonlinear fully connected (FC) layer with metric learning layer (Fig. 2). The nonlinear FC layers comprise the following sequential layers: a linear layer, dropout layer, rectified linear unit (ReLU) activation function, and linear layer. Linear function applies a linear transformation to input matrices. ReLU is an activation function that renders the network nonlinear and fits complex data. Dropout is a regularization method for reducing overfitting in neural networks during training. Furthermore, we introduced AdaCos [59], which is a deep metric learning technique, to control dimensionality reduction complexity while preserving the individual characteristics of each patient. AdaCos [59] can automatically determine hyperparameters and successfully perform deep metric learning without additional tuning steps.

## Similarity Index by the Proposed Model

The cosine similarity between the features in the follow-up and baseline chest X-ray images was used as the similarity index. The cosine similarity ranged from − 1 to 1 and was used for distinguishing same or distinct matched patient pairs. Values closer to 1 present the same patient pair. The cosine similarity is considered superior to the distance measurement in face verification [60].

## Hyperparameter Evaluation of the Proposed Model

Several hyperparameter settings of the proposed method were evaluated in terms of each top-1 accuracy on the PadChest dataset divided into the following four subsets in terms of view position combinations at the baseline and each follow-up examination: PA view at both examinations (PA–PA), AP view at the baseline and PA view at follow-up examinations (AP–PA), PA view at the baseline and AP view at follow-up examinations (PA–AP), and AP view for both examinations (AP–AP).

In this study, we evaluated the following hyperparameters: number of epochs and number of features in the feature extractor output. The number of features was varied by further applying a linear transformation to the output from the feature extractor.

## Verification Performance Evaluation of the Proposed Method

The image with the oldest examination date in the PadChest, YU, and ML datasets was used as the baseline image, and the other images were used as follow-up images. To evaluate the verification performance, this study used all image pairs from the same patient and the image pairs from different patients by random sampling: PadChest (6376 and 6376 of respective same and different patient pairs), YU (4100 and 4100 of respective same and different patient pairs), and ML (18,576 and 18,576 of respective same and different patient pairs) datasets. For the CheXpert dataset, we used the image-pair list, of which 8243 were of same patient pairs and 8243 of different patient pairs, used in a previous study [12].

The verification performance of the proposed method was evaluated in terms of the area under the receiver operating characteristic (ROC) curve (AUC) and equal error rate (EER). The ROC curves were statistically compared using the unpaired DeLong’s test [61].

## Closed-Set Identification Performance Evaluation of the Proposed Method

The image with the oldest examination date in the PadChest, YU, and ML datasets was used as the baseline image, and the other images were used as the follow-up image by employing all combinations for closed-set identification performance evaluation: PadChest (baseline 6,376 images of 6376 patients and follow-up 13,337 images of 6376 patients), YU (baseline 12,517 images of 12,517 patients and follow-up 13,030 images of 4100 patients), and ML (baseline 31,366 images of 31,366 patients and follow-up 49,743 images of 18,576 patients) datasets.

The closed-set identification performance of the proposed method was evaluated in terms of top-1 and top-2 accuracies. Top-2 accuracy refers to the accuracy when any of the pairs within the top-two higher cosine similarities in all patient comparisons are the same patient pairs. The top-1 accuracy on each subset by view position combinations was compared statistically using the two proportion *Z*-test.

## Results

**Fig. 3:**
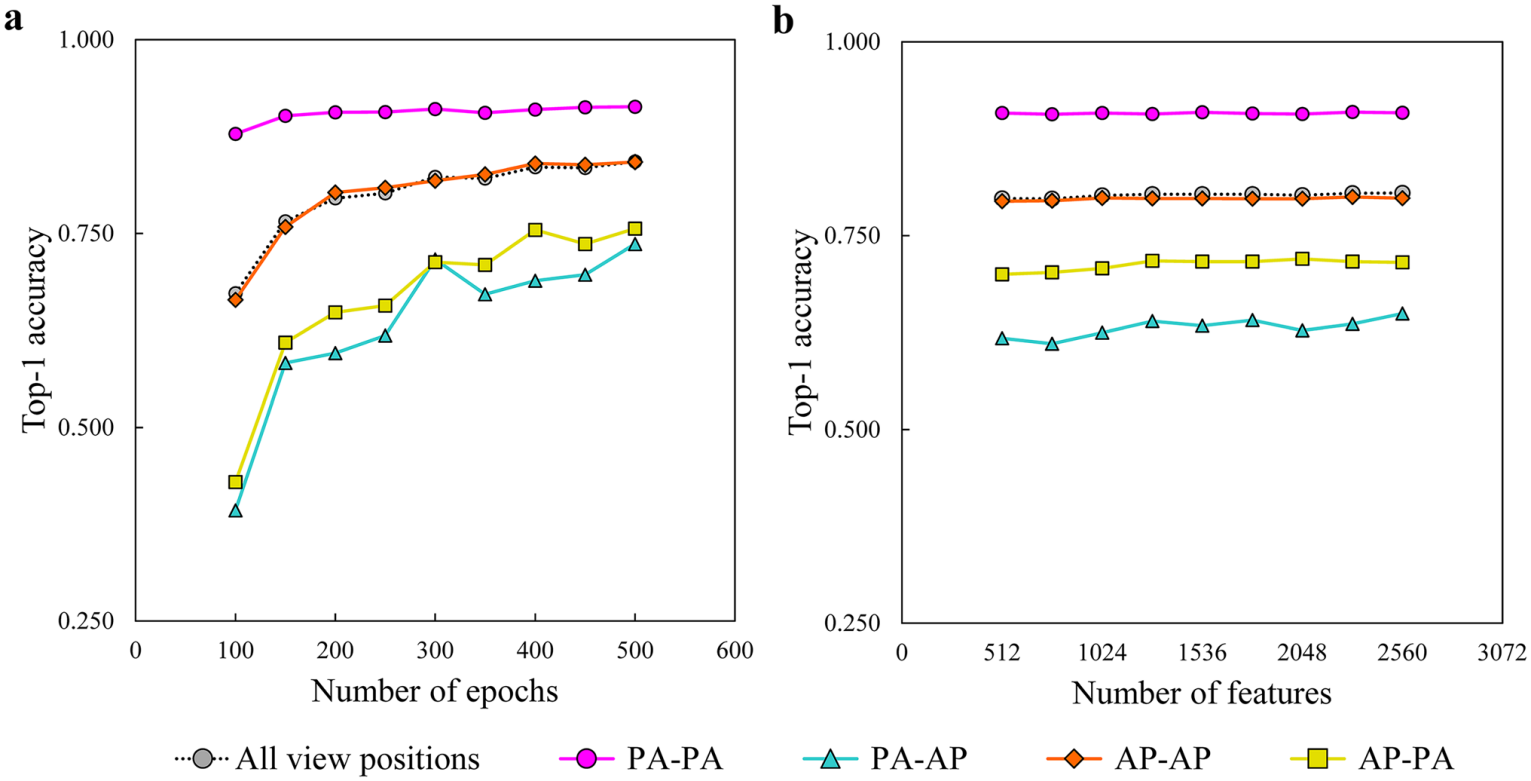
Hyperparameter transition in the proposed method on the PadChest dataset. Two hyperparameters, number of epochs, and number of features in the feature extractor output variabilities show **a** the relationship between the number of epochs and top-1 accuracy, **b** the relationship between the number of features and top-1 accuracy. PA–PA subset for PA view at both examinations, PA–AP subset for PA view at baseline and AP view at follow-up examinations, AP–AP subset for AP view for both examinations, AP–PA subset for AP view at baseline and PA view at follow-up examinations

Figure 3 shows the top-1 accuracy transition of the hyperparameter variabilities on the PadChest dataset and four subsets, which are PA–PA, PA–AP, AP–PA, and AP–AP, using the proposed method. As can be observed in Fig. 3a, the performance of the proposed method improved with the increase in the number of epochs to 300. As shown in Fig. 3b, the top-1 accuracy transition remains flat regardless of the number of features.

**Fig. 4:**
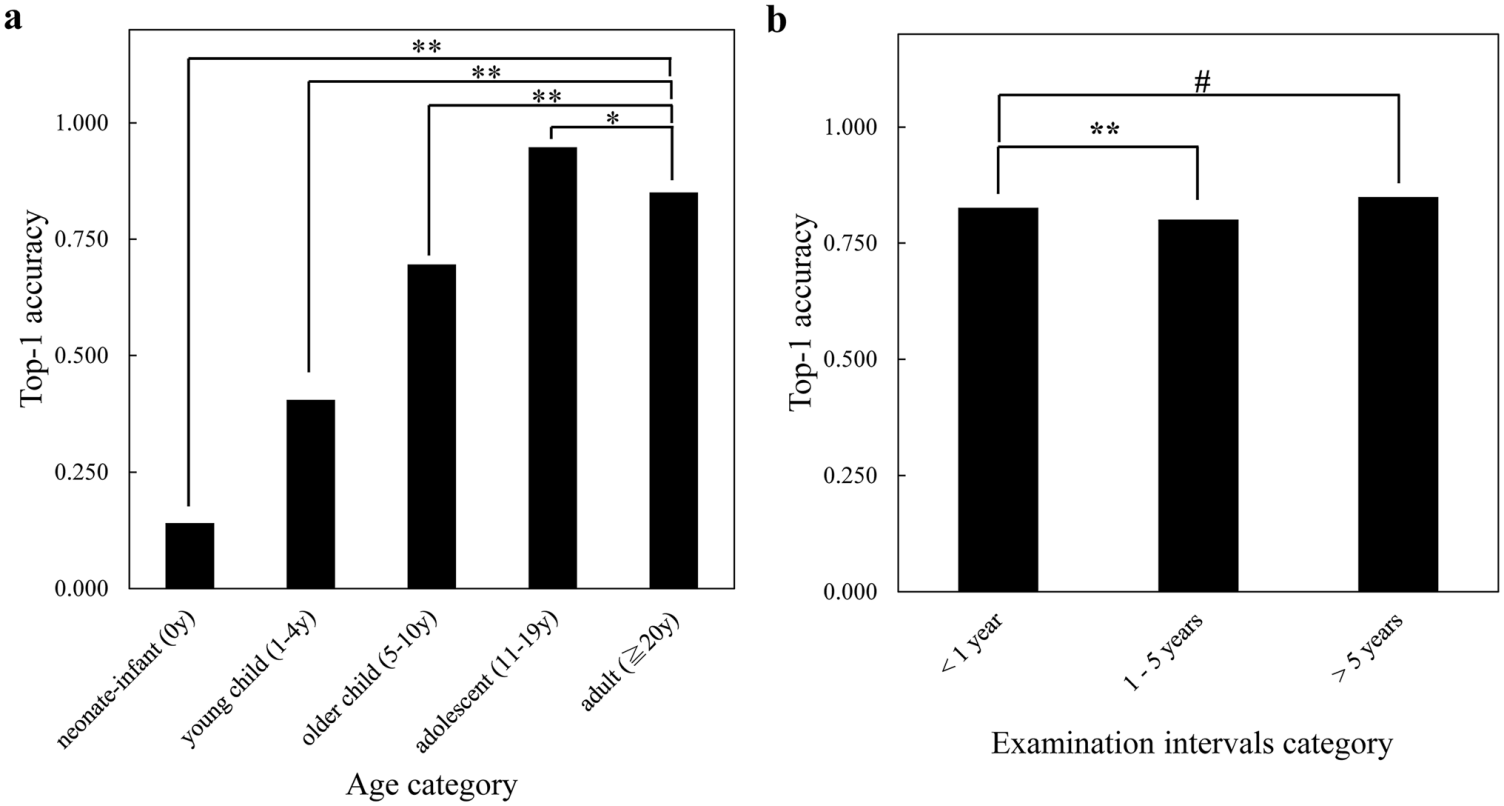
Top-1 accuracy performance using the proposed method on the PadChest dataset. Two categories, age and examination intervals, variabilities show **a** the relationship between the age category subsets and top-1 accuracy, **b** the relationship between the examination intervals category subsets and top-1 accuracy. Neonate-infant: subset under the age of 0 years; young child: subset aged between 1 and 4 years; older child: subset aged between 5 and 10 years; adolescent: subset aged between 11 and 19 years; adult: subset aged 20 years or more. < 1 year: subset in which the examination interval is less than 1 year, 1–5 years: subset in which the examination interval is between 1 and 5 years, > 5 years: subset in which the examination interval is 5 years or more. Asterisks: significant difference (two proportion *Z*-test; *p < 0.05 and **p < 0.01). Pound: no significant difference (two proportion *Z*-test; #*p* > 0.05)

Figure 4 shows the top-1 accuracy transition of (a) patient age in the baseline examination and (b) examination interval between the variabilities in the baseline and follow-up categories on the PadChest dataset using the proposed method with the result of the two proportion *Z*-test. In Fig. 4a, it can be observed that the top-1 accuracy increased until the age category reached adolescent. The top-1 accuracy of the adult category was significantly lower than that of adolescent (*p* < 0.05). Furthermore, in Fig. 4b, it is seen that the top-1 accuracy with an examination interval of less than one year was not significantly different from those with examination intervals from one to five years and greater than five years.

Table 2 summarizes the verification performance of the proposed method in terms of AUC and EER values on two public and two clinical datasets with 300 epochs and 1,280 features. Significant differences were observed between each different dataset (*p* < 0.01). The AUC values of the proposed method on the CheXpert (AUC = 0.9943) and ML datasets (AUC = 0.9999) were superior to those of the methods proposed by Packhäuser et al. on the CheXpert dataset (AUC = 0.9870) [12], Morishita et al. on the ML dataset (AUC = 0.993) [16], and Shimizu et al. on the ML dataset (AUC = 0.994) [17].

**Table 2:** Verification performance of the proposed method on two public and two clinical datasets

Dataset	EER	AUC	p value		
			vs. CheXpert	vs. ML	vs. YU
PadChest	2.70 × 10^−2^	0.9899	2.02 × 10^−7^	2.20 × 10^−16^	2.20 × 10^−16^
CheXpert	2.96 × 10^−2^	0.9943	–	2.20 × 10^−16^	2.98 × 10^−6^
ML	5.65 × 10^−4^	0.9999	–	–	9.10 × 10^−4^
YU	5.12 × 10^−3^	0.9980	–	–	–

*p* value calculated for AUC values by the unpaired DeLong test. *ML* Morishita Laboratory, *YU* Yamaguchi University, *EER* Equal Error Rate, *AUC* Area under the receiver operating characteristic curve

Table 3 summarizes the closed-set identification performance of the proposed method in terms of top-1 and top-2 accuracies on a public and two clinical datasets with 300 epochs and 1280 features. The closed-set identification performance of follow-up examinations with different view positions (PA–AP or AP–PA) was significantly lower than that with a same view position (PA–PA or AP–AP). Furthermore, although the top-2 accuracy increased to 0.984, the top-1 accuracy showed a sharp decrease to 0.749 on the YU dataset.

**Table 3:** Identification performance of the proposed method on a public and two clinical datasets

Dataset	Subset	Number of patients	Top-1 accuracy	*p* value	Top-2 accuracy
PadChest	PA–PA	3,584	0.910	1.42 × 10^–68^	
	PA–AP	1,412	0.717		
	AP–AP	7,184	0.818	1.01 × 10^–16^	
	AP–PA	1,157	0.713		
	All view positions	13,337	0.823		0.875
ML	PA–PA	49,743	0.993		0.999
YU	PA–PA	11,487	0.738	0.742	
	PA–AP	613	0.731		
	AP–AP	624	0.923	1.23 × 10^–9^	
	AP–PA	306	0.781		
	All view positions	13,030	0.747		0.981

*p* value calculated for top-1 accuracy by the two proportion *Z*-test. *ML* Morishita Laboratory, *YU* Yamaguchi University, *PA–PA* subset for PA view at both examinations, *AP–PA* subset for AP view at baseline and PA view at follow-up examinations, *PA–AP* subset for PA view at baseline and AP view at follow-up examinations, *AP–AP* subset for AP view for both examinations

**Fig. 5:**
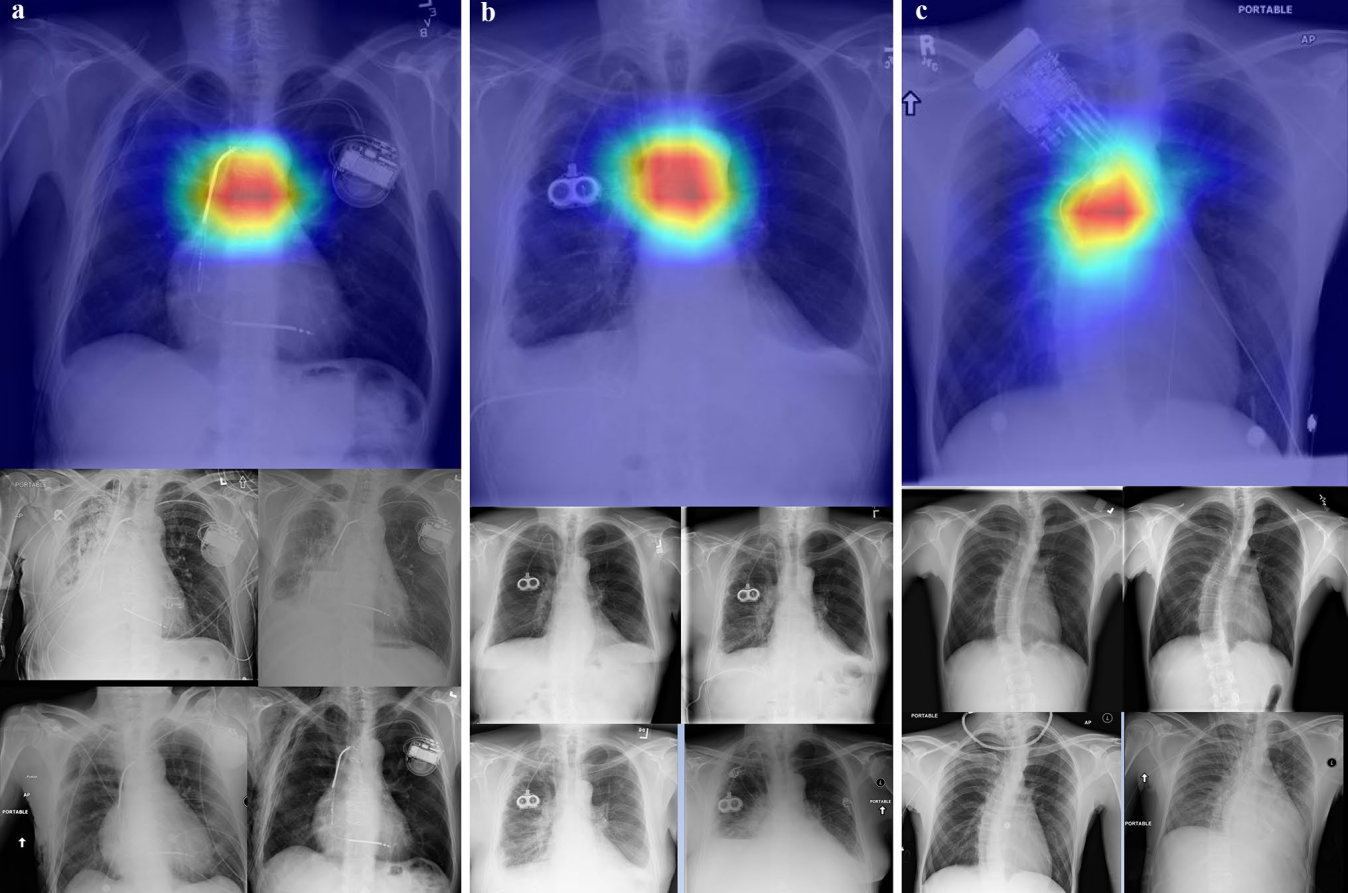
Visualization of an important area that identifies the patient with the trained model of the proposed method using Grad-CAM. Example images of three patients (**a**–**c**) with five images each, one image from the validation dataset and four images from the training dataset. **a**–**b** Example image of patients with an implanted device (**a** pacemaker and **b** CV port). **c** Example image of a patient with temporary devices (some devices are in the training image and the other is in the validation image). The validation images overlayed the Grad-CAM [62] map to visualize the parts of an image that are important for patient identification using the proposed method. The patients in the first and second rows are different

Figure 5 shows an important area visualization that identifies the patient using the model trained based on the proposed method by Grad-CAM [62]. Grad-CAM generates heat maps of characteristic features and superimposes them on the original X-ray image to visualize the important regions that affect patient verification.

## Discussion

In this study, a DCNN feature extractor and classifier based on the similarity index was used to verify the identity of examined patients using clinical chest X-ray images. The proposed method is available for use not only from the second examination onwards without additional training but also extracts robust features regarding different view positions. The number of features output by the EfficientNetV2-S backbone is used as a hyperparameter in the proposed method. We set the number of features to 1280 and the number of epochs to 300. The results revealed that this evaluation method achieved high performance not only on public datasets containing a large amount data and different view positions but also on clinical datasets that include clinical image variabilities (Tables 2–3). Packhäuser et al. [12] achieved an AUC value of 0.9870 on the CheXpert dataset. Furthermore, Morishita et al. [16] and Shimizu et al. [17] achieved the highest AUC values of 0.993 and 0.994, respectively, on the ML dataset. In comparison, the proposed method achieved AUC values of 0.9999 and 0.9943 on the ML and CheXpert datasets, respectively, which are both higher than those achieved by the previous methods [12, 16, 17].

In the comparison (PA–AP and AP–PA subsets in the PadChest dataset) between the combinations of different view positions shown in Fig. 3a, the top-1 accuracy improved when the number of epochs was increased to 300. The results in the PA–PA and AP–AP comparisons, that is, combinations of the same view position, were sufficiently accurate even with 200 epochs, and increasing the number of epochs did not improve the accuracy. Therefore, the optimal number of epochs depends on the type of view positions present in the dataset. These results suggest that underfitting may occur in feature extraction at different view positions up to 300 epochs. More than 300 epochs are required to extract robust features for different view positions. We assume that, depending on the model, when the number of epochs exceeds 300 during training, techniques such as regularization and data augmentation are needed to suppress overfitting in the proposed method.

An increase in the interval between two examinations negligibly affected the top-1 accuracy of the proposed method, as shown in Fig. 4b; therefore, the examination intervals are expected to not be problematic for clinical applications of this method.

Areas around the devices implanted in the three patient examples shown in Fig. 5 are hardly recognized as important areas for feature extraction. Although we are concerned that the devices might be recognized as characteristic features, we believe that the proposed method has the potential to be applied to patient verification and identification from chest X-ray images even if high-contrast objects, such as accessories (removable) or metal implants (not removable), are present in the imaging area. Furthermore, changes in lung condition due to pneumonia or slightly oblique patient positions do not affect the performance of the proposed method in extracting patient-characteristic features.

This study has certain limitations. First, the results suggest that patient identification using chest X-ray images has a domain-shift problem in different view positions, including the reproducibility of the positioning. The performance of the proposed method on the ML dataset was consistently superior to that on the YU, PadChest, and CheXpert datasets in terms of AUC, EER, and top-1 and top-2 accuracies, likely because all patients in the ML dataset underwent the chest X-ray imaging in a standing or sitting PA view position. In terms of the reproducibility of positioning, the ML dataset, which does not include the AP view position, is comprehensively superior to the YU, PadChest, and CheXpert datasets. Furthermore, results revealed a degradation in the top-1 accuracy performance between different view positions (AP–PA or PA–AP) compared with images from the same view position (PA–PA or AP–AP). We demonstrated that one of the solutions to this problem is to increase the number of epochs and suppress underfitting. Although the performance on the YU, PadChest, and CheXpert datasets was not as effective as that on the ML dataset, the metrics showed reasonably good results. However, there is a statistically significant difference between the top-1 accuracies for the same and different view positions. We expect further accuracy improvements using applications such as domain adaptation techniques [12, 28].

Second, in Fig. 4a, significant differences can be observed in the performance of the proposed method in terms of the top-1 accuracies for each patient age category. The top-1 accuracies were significantly lower for the neonate-infant, young child, and older child categories compared with the adult category. We concluded that the performance degradation was because the model underfitted to the changes in the patient’s body over the growth period, which could be attributed to no images of patients aged under 20 in the training dataset. Further research is required on efficient learning with small datasets.

Third, although the top-2 accuracy increased to 0.984, the top-1 accuracy showed a sharp decrease to 0.749 on the YU dataset. At the hospital that provided the YU dataset, a new patient identifier (ID) is issued when a patient visits for the first time; however, in some cases, an already registered patient with a patient ID was assigned a new patient ID. In this case, two patient IDs were assigned to the same patient. In this study, the patient ID registered in the DICOM header was treated as one patient with one ID. Therefore, if the patient ID recorded in the DICOM header is different, it will be regarded as the image of a different patient, even if it is the same patient. However, if the similarity is high because it is the same patient, the top-1 accuracy still degrades. Furthermore, the YU dataset contains some inadequate X-ray images resulting from errors that occur while reading and transferring the images. However, the patient ID is correctly recorded in the header of these images. Although it is the same patient on the ID recorded in the DICOM images, the results show that the similarity index is low between inadequate and other adequate images. A record that has been assigned two IDs is managed in the hospital information system (HIS) or radiology information system (RIS). It is, therefore, desirable to use the patient IDs recorded in HIS/RIS information, not the patient IDs recorded in DICOM images, for lists used in biometric systems in clinical applications. Moreover, we believe that measures to disable inadequate images presented above are desirable.

In addition, in this study, each clinical dataset was obtained using X-ray imaging devices with the same model and similar acquisition parameters. Different X-ray imaging devices have different image contrast in chest X-ray images; therefore, the performance achieved in this study may not be reproducible. To evaluate the biometric performance on a variety of image contrasts, a dataset consisting of clinical X-ray images from several clinics or hospitals using common patient identifier is required.

The proposed method can confirm that the patient who underwent chest X-ray examination and the patient to be examined are the same. Moreover, the proposed method is available for checking the misfiling patient identifier on the picture archiving and communication system. We thereby expect that the proposed method will be useful for safety systems that could prevent these patient misidentification problems and will be able to contribute to the reduction of the risk of incorrectly identifying patient due to human error.

## Conclusions

Potential risks that can lead to medical malpractice, such as patient misidentification in clinical examinations and misfiling of images stored in picture archiving and communication systems, are still inherent in clinical work. In this study, a patient verification and identification method was proposed using a deep feature extractor with robustness under different view positions from images obtained by clinical chest X-ray examination. The performance of the proposed method was verified not only on two public datasets but also on two clinical image datasets, namely ML and YU. Clinical chest X-ray examinations that utilize cutting-edge technology (such as the technique presented in this study) provide a key solution to prevent patient misidentification resulting from human errors.

## Data Availability

All chest X-ray images in this study were compiled from three public and two clinical datasets. ChestX-Ray8 published by Wang X et al. [50] is available in the ChestXray-NIHCC repository at https://nihcc.app.box.com/v/ChestXray-NIHCC/. PadChest published by Bustos A et al. [51] is available in the PadChest repository at https://bimcv.cipf.es/bimcv-projects/padchest/. CheXpert published by Irvin J et al. [52]. is available in the CheXpert repository at https://stanfordmlgroup.github.io/competitions/chexpert/. Two clinical datasets, ML and YU, are owned by the Morishita Laboratory in Kyushu University, Japan, and Yamaguchi University Hospital, Japan, respectively. ML and YU cannot be made publicly available owing to patient privacy, its proprietary nature, and ethical concerns.

## Code Availability

The code necessary to reproduce the results achieved by the proposed method on public datasets can be found at https://github.com/d83yk/dml-cxr-patient-identification.
